# The impact of working time conditions on nurses’ private life: a cross-sectional study on spillover effects and structural factors for recovery

**DOI:** 10.1186/s12912-026-04519-w

**Published:** 2026-03-05

**Authors:** Pia Brants, Susanne Hanefeld, Ann-Sophie Jacobs, Jasmin Hüttl, Sabrina Hanika, Katrin Lücker, Denise Vey

**Affiliations:** 1 Fachbereich Gesundheit, Technische Hochschule Mittelhessen, Willy Robert Pitzer-Institut für Versorgungsforschung und Rehabilitation, Wiesenstraße 14, Gießen, 35390 Deutschland; 2 Technische Hochschule Mittelhessen, Fachbereich Gesundheit, Gießen, Deutschland

**Keywords:** Nursing staff, Work-life balance, Recovery, Shift work, Working time

## Abstract

**Background:**

Working conditions in the nursing profession, characterized by shift work and staff shortages, severely impair nurses’ capacity for recovery. This leads to a negative spillover effect, where work-related stress and exhaustion impact private life, threatening staff well-being and retention. While the consequences are known, the specific structural and organizational drivers of these recovery deficits are not fully understood. This study aims to identify key structural factors in working time arrangements that impact nurses’ recovery.

**Methods:**

A quantitative, cross-sectional online survey was conducted between August and October 2024. The sample included 544 nursing professionals from various stationary care sectors in Germany. A standardized questionnaire, developed from validated scales, assessed working time arrangements (overtime, breaks, rest periods), schedule predictability, and negative spillover effects from work to private life. Data were analyzed using descriptive statistics, Spearman’s correlations, and a multiple linear regression model.

**Results:**

The findings revealed a high prevalence of structural stressors, with a majority of nurses reporting regular overtime (61.5%), frequently missed breaks (61.2%), and non-adherence to statutory rest periods (55.9%). A multiple linear regression analysis, explaining 27.8% of the variance in negative spillover, showed that all five investigated structural factors were significant, independent predictors. The frequent omission of breaks emerged as the strongest unique predictor, followed by the frequency of schedule changes, schedule unpredictability (lead time), overtime, and insufficient rest periods.

**Conclusion:**

This study provides strong evidence that nurses’ recovery is critically dependent on structural organizational frameworks. Factors such as overtime, insufficient recovery periods, and schedule instability are key, modifiable drivers of the negative spillover effect, which undermines nurses’ work-life balance. Healthcare institutions and policymakers must shift from focusing on individual coping to implementing robust organizational policies. Key strategies include enforcing statutory rest periods, establishing predictable scheduling, and investing in staffing models that prevent systematic overtime. These structural interventions are essential to improve nurse retention and ensure the long-term sustainability of the nursing workforce.

**Trial registration:**

Clinical trial number: not applicable.

## Background

The working conditions in the nursing profession are a global concern, posing a significant risk to the stability of healthcare systems worldwide. Nursing staff are exposed to a multitude of work-related stressors, including high physical and psychological demands, staff shortages, and a growing care burden, which severely limit their capacity for recovery [1]. A key challenge is the prevalence of 24/7 shift work, which disrupts the natural sleep-wake cycle and significantly impairs nurses’ ability to recover [2].

These demanding conditions are reflected in high rates of sickness absence. In Germany, for example, nurses were on sick leave for an average of 28.5 days in 2024 [3], considerably higher than the general workforce average of 15.1 days [4]. This issue is not unique to Germany, as international studies from the UK, USA, and Australia report similar trends of high workload and burnout among nursing staff [5–7]. Insufficient breaks, non-compliance with statutory rest periods, irregular shift patterns, and a lack of schedule predictability often lead to chronic overload, with negative consequences for nurses’ health, job satisfaction, and retention in the profession [8]. This situation contributes to a high turnover rate and an estimated training dropout rate of 25% in Germany, as existing working conditions are often perceived as incompatible with personal life aspirations [9]. Projections indicate a significant shortfall of nursing staff in many Western countries, with Germany facing a potential deficit of up to 690,000 nurses by 2049 [10].

A crucial factor in retaining nursing staff is the ability to achieve a healthy work-life balance (WLB), which is defined as the successful management of work and private life domains [11]. To understand how specific work arrangements disrupt WLB, the Effort-Recovery (E-R) Theory [12] and the Boundary/Spillover Theory [13, 14] provide an essential theoretical framework. The E-R Theory posits that work-related effort depletes psychophysiological resources that must be replenished during recovery periods to prevent chronic strain [12]. Building on this, the Boundary/Spillover Theory explains how negative states, such as exhaustion from work, can cross the boundary into private life, a process known as negative spillover [13, 14]. This spillover can be time-based, where work scheduling conflicts with private obligations and leisure, or strain-based, where work-related exhaustion and stress impair private life [15]. If work conditions systematically prevent the necessary recovery described by the E-R model, a negative spillover is likely to occur, directly impairing an individual’s WLB.

This study focuses on four specific structural working-time arrangements hypothesized to impede these recovery processes. In this context, overtime is defined as any working time that exceeds the contractually agreed-upon hours. Missed breaks refer to the failure to take statutory rest periods during a shift. Insufficient rest periods describe non-adherence to the legal minimum rest time between two consecutive shifts. Lastly, schedule instability encompasses short-term, unforeseen changes to the duty roster that hinder the reliable planning of private life and recovery phases.

While the detrimental effects of factors like overtime and staff shortages are well-documented, in international research a clear understanding of how these specific, structural working-time arrangements drive the negative spillover effect and hinder recovery is still lacking [16, 17]. Many studies focus on individual coping mechanisms [18], but there is a research gap regarding the organizational and structural levers that could systematically improve nurses’ recovery capacity and thereby enhance their WLB [17, 19].

Grounded in this theoretical framework, this study aims to address this gap by analyzing the relationship between working time-related factors and the impairment of WLB (via the spillover effect) among nurses in the stationary care sector. The objective is to identify key structural factors in working time arrangements that significantly impact nurses’ recovery, thereby providing evidence-based insights for improving work conditions and promoting staff retention.

## Study hypotheses

Based on the existing literature, the following hypotheses were formulated:

### H1

A higher amount of overtime is positively associated with negative spillover.

### H2

Insufficient recovery periods, specifically (a) the frequent omission of breaks and (b) non-adherence to statutory rest periods, are positively associated with negative spillover.

### H3

Greater schedule instability, specifically (a) a higher frequency of schedule changes and (b) lower predictability (i.e., shorter lead times), is positively associated with negative spillover.

## Methods

### Study design

To explore the structural factors impacting nurses’ work-life balance and recovery, a quantitative, cross-sectional survey design was utilized [20].

### Setting and participants

The study targeted German-speaking nursing staff working in the stationary healthcare sector in Germany. This included staff from hospitals, inpatient nursing homes (short- and long-term care), preventive care and rehabilitation facilities, psychiatric centers, and hospices [21]. Participants from all sociodemographic backgrounds were included. Staff working in ambulatory (outpatient) care were excluded from the study.

### Sample size calculation

An a-priori power analysis was conducted using G*Power (Version 3.1.9.7) to determine the minimum required sample size for the main multiple linear regression analysis. Assuming a medium effect size (f² = 0.15), an alpha level of 0.05, a statistical power of 90%, and five predictors in the model, the required sample size was determined to be *N* = 138. The final achieved sample of 544 participants comfortably exceeded this minimum, ensuring sufficient statistical power for all regression analyses [22].

### Data collection and instrument

The questionnaire was specifically compiled for this study by integrating items from several established and validated scales, rather than being a single pre-existing instrument. Data were collected using a standardized online questionnaire developed in MS Forms. The questionnaire was structured into five thematic sections. For the purposes of this study, three sections were included in the analysis, focusing on working time arrangements, spillover effects on private life, and sociodemographic information. The order of items was fixed and not randomized. While completion of all items was encouraged, it was not mandatory, allowing participants to skip questions. A back button was provided to change previous answers. To enhance usability and reduce participant burden, these 44 questions were distributed across approximately 14 distinct screens. Items were grouped thematically on these pages.

The instrument was developed based on several established and validated scales. The section on working time arrangements was based on items from the German Federal Institute for Occupational Safety and Health (BAuA) Working Time Survey 2021 [23]. The variable Overtime was operationalized as the metric difference between participants’ self-reported actual average hours and their contractually agreed weekly hours. The overarching concept of Insufficient Recovery Periods was captured through two separate binary items. One assessing the frequent omission of statutory breaks during long shifts, and another identifying non-adherence to the legal 11-hour rest period between shifts. Finally, the multifaceted construct of Schedule Instability was operationalized using two key items. The frequency of changes was assessed by asking how often work schedules were altered due to operational requirements, with responses ranging from “frequently” to “almost never”. The predictability of these changes was measured based on the typical lead time participants received, with responses categorized from “on the same day” to “more than two weeks in advance,” and importantly, including a separate category for “unpredictable” lead times [23].

Negative spillover was measured using the German version of the “Negative Spillover from Work to Private Life” scale (B-AOF) by Schuller and Rau. Based on pre-test results indicating low reliability for the private-to-work direction (Cronbach’s α = 0.390), only the subscales measuring the work-to-private-life direction (8 items, covering time-based and strain-based spillover) were used [15]. For the analysis, a total mean score for negative spillover was calculated, an approach supported by the high correlation between the time-based and strain-based subscales (*r* = .70) reported in the original validation study by Schuller and Rau [15]. The pre-test confirmed good to acceptable reliability for the overall work-to-private life scale (Cronbach’s α = 0.828) and its subscales for strain-based spillover (α = 0.766) and time-based spillover (α = 0.737). Finally, standard sociodemographic variables such as age, gender, marital status, and household size were collected [23].

The instrument’s clarity and validity were ensured through a three-stage pre-testing process. An initial self-test by the researchers, an expert review with 11 subject matter and methodology experts, and a field pre-test with 20 participants from the target population [20, 24]. Feedback from these pre-tests led to minor refinements of question wording, layout, and technical functionality.

### Procedure

Recruitment took place between August 7th and October 18th, 2024. A non-probability, convenience sampling approach was used [25]. Participants were recruited passively through self-selection via flyers distributed on social media platforms in groups specific to nursing professionals [26, 27]. The flyers contained a short description of the study, a QR code, and a link to the online survey. Additionally, a snowball sampling technique was employed, as participants were encouraged to share the survey link within their professional networks [27]. Participation was fully anonymous. As a non-monetary incentive, participants registered on a research platform could receive points for their participation.

### Ethical considerations

Informed consent to participate was obtained from all participants. The study was conducted in accordance with the principles of the Declaration of Helsinki. An introductory page to the survey informed participants about the study’s purpose, the voluntary nature of their participation, and the anonymous handling of their data according to data protection regulations [28]. Consent was implied by the completion and submission of the questionnaire. The need for formal ethics approval was waived by the Technische Hochschule Mittelhessen, as the research did not involve interventions or sensitive personal data beyond the scope of standard occupational surveys and all participation was fully anonymous and voluntary.

### Statistical analysis

Data was exported from MS Forms to MS Excel for initial cleaning and then to IBM SPSS Statistics (Version 29) for analysis. To prevent multiple entries from the same individual, the dataset was screened for duplicate or suspicious response patterns, but none were identified; the survey platform itself used browser cookies to discourage repeat participation. Out of 754 initial responses, 210 were excluded because they did not meet the inclusion criteria (*n* = 41 not working in healthcare, *n* = 169 working in ambulatory care), resulting in a final dataset of *n* = 544. The proportion of missing data within the final eligible sample was 3.15%.

Descriptive statistics (frequencies, means, standard deviations) were used to characterize the sample and variables [29]. The main inferential analysis followed a two-step hierarchical approach. First, to examine the bivariate relationships between the structural working time arrangements and negative spillover, Spearman’s rank correlation coefficient (rs) was calculated. Second, to provide a robust test of the study hypotheses, a multiple linear regression analysis was conducted. This method allowed for testing the unique contribution of each factor while statistically controlling for all other variables. The five structural working time variables were entered simultaneously as independent variables to predict the total negative spillover score. Key assumptions of the regression model, such as multicollinearity (assessed via the Variance Inflation Factor, VIF), were checked. A significance level of *p* < .05 was set for all analyses. No statistical corrections, such as sample weighting or propensity scores, were applied to adjust for the non-representative nature of the convenience sample. For the multiple regression analysis, cases with missing data on any of the included variables were excluded listwise, resulting in a final sample of *N* = 511 for this specific analysis.

## Results

### Sample characteristics and descriptive statistics

A total of 544 nursing professionals participated in the survey. Table 1 shows that the sample consisted predominantly of female (76.9%, *n* = 416), with the largest age group being 30–39 years (39.8%, *n* = 214). The majority of respondents worked in hospitals (56.4%, *n* = 307), followed by in-patient nursing homes (28.3%, *n* = 154) and other stationary facilities (15.3%, *n* = 83).

**Table 1:** Sample characteristics (*N* = 544)

Characteristic	Category	*N*	%
Work Setting	Hospital	307	56.4
	In-patient Nursing Home	154	28.3
	Other stationary facility	83	15.3
Gender	Female	416	76.9
	Male	123	22.7
	Diverse/Other	2	0.4
Age Group	< 20 years	6	1.1
	20–29 years	122	22.6
	30–39 years	214	39.8
	40–49 years	131	24.3
	50–59 years	52	9.7
	≥ 60 years	8	1.5
Employment Type	Full-time	290	53.7
	Part-time	233	43.1
	Other (e.g., in training)	17	3.2

The descriptive analysis of the structural working time stressors revealed a high prevalence across the sample. Regarding overtime, the actual working hours significantly exceeded contractually agreed hours. Full-time nurses reported working an average of 42.86 h per week against a contractual average of 39.09 h, resulting in a mean weekly overtime of approximately four hours. This aligns with national data on overtime in the nursing sector [30]. Overall, 61.5% (*n* = 320) of all participants reported working overtime regularly. This issue was prevalent across different employment arrangements.

Insufficient recovery periods were also widespread. A majority (61.2%, *n* = 329) stated that their scheduled breaks were frequently missed during shifts longer than six hours. Of this group, more than half received no compensation for the missed time. Furthermore, 55.9% (*n* = 302) of participants reported that the legally mandated rest period of 11 h between shifts was not consistently adhered to, with 5.6% (*n* = 17) of this subgroup experiencing this issue almost every working day.

In terms of schedule instability, a large majority of participants (78.1%, *n* = 418) reported that short-notice changes to their work schedule occurred “frequently” or “occasionally.” The predictability of these changes was low, with half of the respondents (50.1%, *n* = 268) stating that the lead time for schedule alterations varied unpredictably, severely hindering the ability to plan private life.

These working conditions were associated with a significant impact on private life. A majority of nurses (69.0%, *n* = 367) experienced regular strain-based negative spillover effects, defined as work-related exhaustion carrying over into their private time. Consequently, 78.3% (*n* = 417) reported having insufficient free time for recovery due to work and family commitments.

To examine the initial relationships between the key study variables, a Spearman’s rank correlation analysis was conducted. The results, presented in Table 2, reveal that all five structural working time arrangements were significantly and substantially correlated with negative spillover.

The strongest bivariate relationship was found for missed breaks, indicating that nurses who frequently missed their breaks reported significantly higher levels of negative spillover (rs = − 0.380, *p* < .001). This was closely followed by the frequency of schedule changes (rs = − 0.370, *p* < .001) and insufficient rest periods between shifts (rs = − 0.303, *p* < .001). Furthermore, a shorter lead time for schedule changes (rs = − 0.270, *p* < .001) and working more overtime (rs = 0.261, *p* < .001) were also associated with higher negative spillover. These results provide initial support for all three study hypotheses.

**Table 2:** Bivariate spearman’s Rho correlations between study variables

Variable	1	2	3	4	5	6
1. Overtime (hours)	—					
2. Schedule Instability (Frequency)	− 0.265**	—				
3. Schedule Instability (Lead Time)	− 0.184**	0.179**	—			
4. Missed Breaks	0.245**	− 0.264**	− 0.177**	—		
5. Insufficient Rest Periods	0.129**	− 0.336**	− 0.128**	0.262**	—	
6. Negative Spillover (Total)	0.261**	− 0.370**	− 0.270**	0.380**	0.303**	—

Note. ** *p *< .01 (2-tailed). Sample sizes (N) for the correlations ranged from 513 to 540 due to pairwise exclusion of missing data. Overtime (hours) is the metric difference between actual and contractual hours. For Schedule Instability (Frequency) and (Lead Time), higher values indicate more stability/predictability. For Missed Breaks and Insufficient Rest Periods, variables are coded 0 = No, 1 = Yes. The signs of the correlation coefficients have been adjusted for intuitive interpretation (e.g., a positive sign indicates that the presence of a stressor is associated with more spillover)

#### Hypothesis testing

To provide a robust test of the study hypotheses, a hierarchical linear regression analysis was conducted. First, five separate simple regression models (“crude models”) were run to assess the individual effect of each structural factor on negative spillover. Second, a multiple linear regression model (“adjusted model”) with all five factors entered simultaneously was calculated to assess the unique contribution of each predictor while controlling for the others. The results for both the crude and adjusted models are presented in Table 3.

The final adjusted model was highly significant, F(5, 505) = 38.91, *p* < .001, and explained a substantial portion of the variance in negative spillover (R² = 0.278). As shown in the adjusted model results in Table 3, all five structural factors remained significant, independent predictors after controlling for the other variables.

The strongest unique predictor was missed breaks (β = 0.249, *p* < .001), followed by the frequency of schedule changes (β = − 0.205, *p* < .001). Schedule unpredictability (lead time; β = − 0.147, *p* < .001), working overtime (β = 0.131, *p* < .001), and insufficient rest periods (β = 0.117, *p* = .005) also made significant, independent contributions to higher levels of negative spillover.

These findings provide strong, independent support for all three hypotheses. Specifically, the significant unique effect of overtime supports H1. The significant independent effects of both missed breaks and insufficient rest periods provide robust support for Hypothesis 2. Finally, the significant contributions of both the frequency and unpredictability of schedule changes confirm H3, demonstrating that each factor has a distinct, detrimental effect. Collinearity diagnostics indicated no issues, with all VIF values well below 2, confirming the stability of the model.

**Table 3:** Simple (crude) and multiple (adjusted) linear regression models predicting negative spillover

Predictor	Crude Model β (Beta)	Adjusted Model β (Beta)	VIF
Overtime (hours)	0.261**	0.131**	1.088
Missed Breaks (Yes = 1)	0.380**	0.249**	1.164
Insufficient Rest Periods (Yes = 1)	0.303**	0.117**	1.175
Schedule Instability (Frequency)	− 0.370**	− 0.205**	1.212
Schedule Instability (Lead Time)	− 0.270**	− 0.147**	1.071

Note: ** *p* < .01, *** *p* < .001. For the adjusted model: R² = 0.278; F(5, 505) = 38.91, *p* < .001. β = standardized coefficient. “Crude Model” betas are the standardized coefficients from five separate simple regressions. The “Adjusted Model” includes all five predictors simultaneously. For Frequency and Lead Time, higher values indicate more stability/predictability

## Discussion

This study investigated the impact of structural working time conditions on the private lives of nursing staff, identifying key drivers of negative spillover and barriers to recovery. The findings confirm that objective working time stressors, such as overtime, missed breaks, insufficient rest periods, and schedule instability, are significantly associated with higher negative spillover effects, thereby impairing nurses’ work-life balance and recovery.

### Interpretation of findings

The results strongly supported all three study hypotheses. The multiple regression model was highly significant and explained a substantial portion of the variance in negative spillover (R² = 0.278). Crucially, all five investigated structural factors emerged as significant, independent predictors, confirming that each contributes uniquely to the impairment of nurses’ private lives.

Supporting H1, the multiple regression analysis demonstrated that overtime is a significant independent predictor of negative spillover (β = 0.131, *p* < .001). This finding is particularly noteworthy as it reveals that a higher amount of overtime contributes to a negative impact on private life even when controlling for other challenging work conditions such as missed breaks, insufficient rest periods, and schedule instability. This suggests that the mere reduction of available personal time is a critical factor, and organizational strategies should prioritize the prevention of overtime rather than solely focusing on its compensation. This aligns with international research indicating that excessive working hours are a primary driver of burnout and turnover intentions in nursing [17].

The findings for H2 were particularly striking and revealed the most powerful predictors of negative spillover. The multiple regression analysis identified the frequent omission of breaks as the single strongest independent predictor in the entire model (β = 0.249, *p* < .001). Additionally, the failure to adhere to statutory rest times also made a significant, unique contribution to higher spillover (β = 0.117, *p* = .005).This highlights a critical organizational failure where the immediate demands of care systematically override the fundamental need for staff recovery [2]. These results are consistent with studies identifying insufficient recovery as a direct precursor to emotional exhaustion and reduced performance [18, 31]. The data therefore suggest that ensuring basic, uninterrupted recovery time is one of the most powerful levers for improving nurses’ well-being.

H3 was also strongly supported, as the multiple regression analysis revealed that both facets of schedule instability were powerful, independent predictors of negative spillover. Specifically, a higher frequency of schedule changes (β = − 0.205, *p* < .001) and a lack of predictability (i.e., shorter lead times; β = − 0.147, *p* < .001) both made significant, unique contributions to higher spillover levels, even after accounting for factors like overtime and missed breaks. This finding supports existing literature emphasizing that unpredictability hinders the ability to plan private life, leading to increased stress and dissatisfaction [32, 33]. Notably, a closer examination of the descriptive data suggests that a lead time of at least two days for schedule changes may be a critical threshold to mitigate these negative effects. This reinforces the importance of moving beyond ad-hoc changes towards more structured, participative scheduling approaches to enhance nurses’ WLB [34, 35].

### Implications for nursing and health policy

The findings of this study have direct and actionable implications for nursing management and health policy, calling for a multi-faceted response focused on structural change. A foundational step is to enforce and monitor basic labor protections, moving beyond the normalization of missed breaks and shortened rest periods. Management should implement policies that guarantee uninterrupted breaks and full adherence to statutory rest times, utilizing digital scheduling tools to monitor compliance and make recovery a measurable performance indicator. This must be complemented by a focus on schedule stability. To mitigate the harmful effects of instability, a key policy should be the implementation of a mandatory minimum lead time for changes, such as two weeks as practiced in some European countries [36]. Furthermore, promoting employee participation in the scheduling process through self-rostering tools can enhance predictability and autonomy, directly improving WLB [37]. The approach to overtime should also shift from compensation to prevention. While fair compensation is necessary, the primary focus should be on investing in structural solutions, including realistic staffing models and the strategic use of flexible staffing pools to manage unforeseen absences [2]. Finally, institutions should explore and promote innovative models like the 4-day work week or other compressed schedules that have shown success in pilot projects by providing longer, consolidated blocks of free time for effective recovery [38].

### Strengths and limitations

This study has several strengths. Its large sample size (*N* = 544) provided sufficient statistical power for the analyses, as confirmed by an a-priori power calculation. The study’s focus on objective, modifiable, structural factors, rather than solely individual characteristics, provides actionable insights for organizational change. Furthermore, the use of validated scales enhances the reliability of the findings. Importantly, the core results regarding the negative impact of overtime, missed breaks, and schedule instability align with the broader literature on occupational health. While the sampling was non-random, key demographic characteristics of the sample, such as the gender distribution (77% female), are largely representative of the German nursing population [39].

However, several limitations must be acknowledged. First, the cross-sectional design does not allow for causal inferences. Second, the reliance on self-reported data may be subject to social desirability bias [40, 41]. Third, the convenience and snowball sampling method limits the generalizability of the findings to the entire German nursing population. This is particularly evident in the sample’s age structure; the largest cohort was 30–39 years old, whereas the German nursing population is considerably older, a discrepancy likely attributable to the online recruitment method [42, 43]. Fourth, the sample included nursing staff from various stationary care settings, such as hospitals, nursing homes, and hospices. While this enhances the breadth of the sample, the present study did not analyze potential differences between these settings. Structural conditions, such as staffing levels, patient acuity, and organizational culture, may vary across these institutions and could potentially moderate the observed relationships. Future research could therefore benefit from a comparative analysis to identify setting-specific challenges and interventions [44]. Despite these limitations, the study provides robust evidence on the critical role of structural working time conditions for nurses’ well-being.

## Conclusion

Recovery in the nursing profession is not primarily an individual responsibility but is largely determined by structural and organizational frameworks. Inadequate recovery periods and schedule instability are significant drivers of negative spillover, undermining nurses’ work-life balance. To ensure a sustainable and resilient nursing workforce, the focus of intervention must shift from expecting individual resilience to building organizationally resilient working time arrangements.

## Data Availability

The cross-sectional study data supporting the conclusion of this article is available from the corresponding author upon reasonable request.

## Abbreviations

ANOVA: Analysis of Variance
BAuA: German Federal Institute for Occupational Safety and Health
B-AOF: Negative Spillover from Work to Private Life
DSGVO: Datenschutz-Grundverordnung (General Data Protection Regulation)
WLB: Work-Life Balance
